# Surface electromyography in the assessment of masticatory muscle activity in patients with pain-related temporomandibular disorders: a systematic review

**DOI:** 10.3389/fneur.2023.1184036

**Published:** 2023-05-03

**Authors:** Liliana Szyszka-Sommerfeld, Magdalena Sycińska-Dziarnowska, Gianrico Spagnuolo, Krzysztof Woźniak

**Affiliations:** ^1^Department of Orthodontics, Pomeranian Medical University, Szczecin, Poland; ^2^Department of Neurosciences, Reproductive and Odontostomatological Sciences, University of Naples “Federico II”, Napoli, Italy; ^3^School of Dentistry, College of Dental Medicine, Kaohsiung Medical University, Kaohsiung, Taiwan

**Keywords:** orofacial pain, temporomandibular disorders, pain-related temporomandibular disorders, surface electromyography, masticatory muscle activity

## Abstract

**Background:**

Temporomandibular disorders (TMD) are a set of painful conditions affecting the orofacial region that are prevalent and constitute the most frequent type of non-dental pain complaint in the maxillofacial area. Pain-related TMD (TMD-P) is characterized by ongoing pain in the masticatory muscles, the temporomandibular joint, or surrounding structures. Due to the multiple factors that contribute to the development of this condition, it can be challenging to accurately diagnose. One of the useful method for assessing patients with TMD-P is surface electromyography (sEMG). The aim of this systematic review was to provide a comprehensive overview of the current scientific literature on the evaluation of masticatory muscle activity (MMA) in individuals diagnosed with TMD-P, through the utilization of sEMG.

**Methods:**

To gather relevant information, electronic databases such as PubMed, Web of Science, Scopus, and Embase were searched using specific keywords including: “pain” AND (“temporomandibular disorder*” OR “temporomandibular dysfunction*”) AND “surface electromyography” AND “masticatory muscle activity.” The inclusion criteria were studies assessing MMA in patients with TMD-P using sEMG. The Effective Public Health Practice Project (EPHPP) Quality Assessment Tool for Quantitative Studies was utilized to evaluate the quality of the studies that were included in the review.

**Results:**

The search strategy identified 450 potential articles. Fourteen papers met the inclusion criteria. Global quality rating for significant part of the articles was weak. Most studies showed greater sEMG activity of the masseter (MM) and temporal anterior (TA) muscles at rest in TMD-P subjects than in the asymptomatic controls, while the MM and TA muscles were less active in the pain-related TMD group compared to the non-TMD group during maximal voluntary clenching (MVC).

**Conclusion:**

There were differences in MMA in the TMD-pain population compared to a healthy control group during various tasks. The diagnostic efficacy of surface electromyography in assessing individuals with TMD-P remains unclear.

## 1. Introduction

Temporomandibular disorders (TMD) are a set of painful conditions that are prevalent and constitute the most frequent type of non-dental pain complaint in the maxillofacial area (1, 2). These conditions are linked to various clinical scenarios that impact the stomatognathic system, which primarily involves the masticatory muscles, the temporomandibular joint (TMJ), and other related structures. TMDs can be manifested by tenderness or pain in the muscles and joints, joint noise, and deviation in mandibular movements (3–7). Pain-related TMD (TMD-P) is characterized by ongoing pain in the muscles of the mandible, the temporomandibular joint, or surrounding structures. This pain can be persistent, recurrent, or chronic (4–6). The temporomandibular pain appears to be relatively common; this condition predominantly affects young and middle-aged adults, as opposed to children or older individuals. Furthermore, it is more prevalent in women, occurring approximately twice as frequently as it does in men (8–11).

The Research Diagnostic Criteria for Temporomandibular Disorders (RDC/TMD) is a highly sophisticated and valuable diagnostic instrument that provides both clinical and research criteria for the accurate evaluation of TMD in both pediatric and adult populations. The RDC/TMD were gradually replaced by the updated Diagnostic Criteria for TMD (DC/TMD) (12, 13). It should be noted that the multifactorial etiology of TMDs can be challenging to accurately diagnose. Therefore, it is crucial to have reliable and effective tools and measures in place to ensure proper evaluation (14–18). In the assessment of TMD, certain instruments can offer valuable quantitative data that may prove useful in a clinical setting (16, 17). Among these instruments, surface electromyography (sEMG) has been widely utilized as a non-invasive tool to evaluate patients with TMD, as well as to analyze the electrophysiological behavior of muscles (6, 18–23). The advantages of using sEMG include its ease of use, accessibility, and non-invasive nature. However, it should be emphasized that sEMG is sensitive to impedance imbalances, which may affect the accuracy and reliability of electromyographic (EMG) assessments (18, 19).

In a recent systematic review Dinsdale et al. (24) studied muscle activity using sEMG in adults with persistent TMD compared to healthy controls. They found that in TMD there are changes in masticatory muscle activity (MMA) that are both task-specific and muscle-specific. However, it is also important what differences in MMA exist between pain-related TMD and asymptomatic healthy controls. Research indicates that subjects diagnosed with TMD-P may modify the activation of their masticatory muscles due to sensorimotor interactions. Pain can alter the formation of action potentials and, perhaps, electromyographic activity (25, 26). Additionally, the presence of pain can result in increased variability in EMG signals, which can undermine the accuracy of sEMG assessments (17). Given these limitations, it is important to note that the use of sEMG as a means of evaluating individuals with TMD, particularly pain-related TMD, remains a subject of debate due to the significant variability in results reported in the literature. As such, the diagnostic efficacy of surface electromyography in assessing this condition has yet to be definitively established (17, 18, 27–29).

The aim of this systematic review was to provide a comprehensive overview of the current scientific literature on the evaluation of masticatory muscle activity (MMA) in individuals diagnosed with TMD-P, through the utilization of sEMG. This paper also aimed to summarize the literature on the diagnostic value of sEMG in diagnosing patients with TMD-P.

## 2. Methods

### 2.1. Search strategy

The systematic review adhered to the guidelines outlined in the “Preferred Reporting Items for Systematic Reviews and Meta-Analyses” (PRISMA) (30).

According to PICO (31), the framework for this systematic review is as follows: Population (P): patients with pain-related temporomandibular disorders; Intervention (I): surface electromyography; Comparison (C): pain-related TMD vs. asymptomatic non-TMD patients; Outcomes (O): changes in masticatory muscle activity and diagnostic utility of sEMG in identifying TMD-P patients. The PICO question was: “Does the masticatory muscle electromyographical (EMG) activity in TMD-P patients differ from that in the healthy non-TMD population? and “Is the surface electromyography useful in differentiating between patients with pain-related TMD and asymptomatic patients?” To gather relevant information, electronic databases such as PubMed, Web of Science, Scopus, and Embase were thoroughly searched using specific keywords including: “pain” AND (“temporomandibular disorder*” OR “temporomandibular dysfunction*”) AND “surface electromyography” AND “masticatory muscle activity.”

The literature search was carried out by two independent reviewers (L.S.S. and M.S.D.), who examined all publications without imposing any temporal restrictions. The final search was performed on December 31, 2022, and encompassed all language versions of the publications.

### 2.2. Eligibility criteria

The inclusion criteria for this review were as follows:

- Study type: observational/interventional studies on the assessment of MMA in patients with TMD-P by surface electromyography, English language;
- Outcome of interest: masticatory muscle activity assessed by sEMG;
- Object of the study: (a) comparison of MMA in TMD-P patients with a healthy population without TMD and (b) evaluation of the diagnostic efficiency of sEMG in diagnosing patients with TMD-P;
- Participants: human subjects.

The exclusion criteria were as follows: ineligible study design; ineligible outcome measure; ineligible population, e.g., studies on TMD patients not related to pain, studies on patients with congenital craniofacial malformations; case reports, reviews, animal studies.

### 2.3. Data extraction

After removing duplicates, all titles and abstracts were read by the first (L.S.S.) and reviewed by the second (M.S.D.) author to identify potentially eligible studies. Subsequently, the full texts of the selected works were scrutinized, and the papers were either qualified or excluded based on the predetermined inclusion and exclusion criteria. Only papers comparing MMA in patients with TMD-P with respect to healthy patients without TMD were included. All ambiguities were resolved through discussions between investigators. The Cohen’s Kappa statistic was performed to measure the agreement between the two authors. The review process involved gathering information related to various aspects of the studies, including study design, participant characteristics, diagnostic criteria employed to diagnose and categorize TMD, outcome measures, such as measurement tools, procedures, and data analysis, as well as principal findings. EMG results were collected from each study by a single reviewer (L.S.S.) and documented in an Excel spreadsheet.

### 2.4. Quality assessment

The Effective Public Health Practice Project (EPHPP, McMaster University, Ontario, Canada) Quality Assessment Tool for Quantitative Studies was utilized to evaluate the quality of the studies that were included in the review (32). This tool assesses various components, such as study design, selection bias, confounders, blinding, data collection methods, and withdrawals, and provides an overall rating of the study as either “strong,” “moderate,” or “weak.” The quality assessment was performed independently by two authors (L.S.S and M.S.D). All ambiguities were resolved through discussions between reviewers. The Cohen’s Kappa coefficient for the agreement between the authors was calculated.

## 3. Results

**Figure 1:**
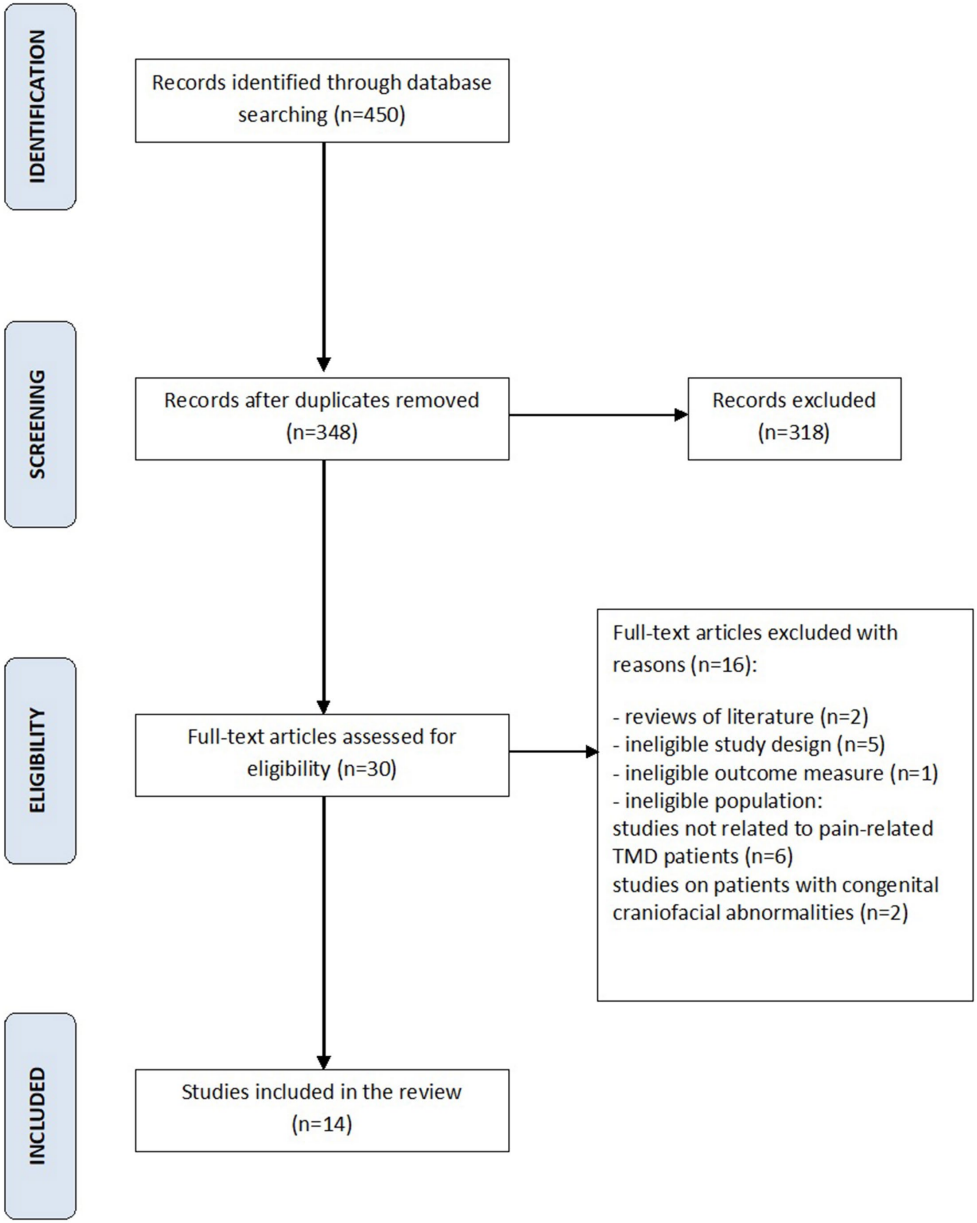
PRISMA flow diagram for the search strategy.

Following search strategy 450 potential articles were identified: 225 from PubMed, 97 from Web of science, 65 from Scopus, and 63 from Embase. After removing of 102 duplicates, 348 articles were analyzed. As a result of title and abstract screening, 318 papers were excluded based on the predetermined inclusion and exclusion criteria. Of the remaining 30 articles, 16 were excluded because they were literature reviews, studies of ineligible study design, studies of ineligible outcome measure, or ineligible population, such as patients with congenital craniofacial anomalies, patients with TMD without pain. Finally, 14 papers were included in the review. The entire process was schematized in the Prisma Flow Diagram (Figure 1, Flow diagram). The Kappa value between two reviewers was calculated as 0.97. Table 1 displays the principal features of each study that was included in the review.

**Table 1:** Characteristics of the study included.

Authors year	Diagnostic criteria	Study groups, participants	Outcomes	Results
Berni et al. 2015 (17)	RDC/TMD	One hundred twenty-three volunteers distributed into 2 groups: myogenous TMD group (80 women diagnosed with myofascial pain (Ia) or myofascial pain with limited mouth opening (Ib); mean age of 23.88 ± 5.53 years) and control group (43 women without TMD; mean age of 22.30 ± 3.18 years)	sEMG evaluation of the TA, MM and suprahyoid muscles were made at rest and during MVC on parafilm.	The TMD group exhibited: significantly greater EMG activity in all muscles at rest, significantly lower activity in the MM muscles and significantly greater potentials in the suprahyoid muscles during MVC on parafilm compared to the control group. Moderate accuracy (AUC: 0.74–0.84) of the sEMG values was found in all muscles regarding the diagnosis of TMD at rest and in the suprahyoid muscles during MVC. Moreover, sensitivity ranging from 71.3 to 80% and specificity from 60.5 to 76.6%.
Bodéré et al. 2005 (33)	Clinical exam	One hundred twelve patients divided into 4 groups: myofascial pain group (n = 33, age 27.4 ± 6.9, ratio 84% female), neuropathic pain group (n = 20, age 43.7 ± 15, ratio 81% female), disc derangement disorders group with non-pain patients (n = 27, age 22.4 ± 7.9, ratio 92% female) and control group of healthy, asymptomatic subjects (n = 32, age 27.1 ± 4, ratio 80% female)	The EMG activity at rest was recorded simultaneously from the left and right MM and left and right TA muscles.	The EMG activity of TA and MM muscles at rest was significantly higher in the pain patient groups compared to the asymptomatic control group. There was no significant difference between the disc derangement disorder group and the control group. In pain patient groups, the increased EMG activity at rest were equally distributed in the pain and non-pain sides.
Glaros et al. 1997 (34)	Clinical exam	One hundred eight individuals: 54 patients diagnosed with myofascial pain (with or without limited opening) (age 25.1 ± 7.6) and 54 non-pain control subjects (age 25.5 ± 7.7). Both groups consisted of 44 women and 10 men.	The EMG activity of the left (L) and right (R) frontalis, temporal and masseter muscles at rest were performed.	The TMD-pain group had significantly higher EMG activity at rest for three of the six sites examined (LTA, LMM, right frontalis). Using the cutoff value that gave the smallest classification error, however, resulted in misclassification of about one third of the TMD and non-pain individuals.
Li et al. 2016 (35)	Clinical exam	Eleven female patients with unilateral TMD pain (11 had myalgia and 3 also had TMJ arthralgia, mean age 23 ± 5.9) and 20 healthy female volunteers (mean age 26 ± 3.2)	The sEMG activity of the TA and MM muscles during MVC in the centric and eccentric positions were simultaneously recorded on both sides.	The sEMG activity of the pain-side TA and bilateral MM was lower during centric MVC compared with controls. During pain-side MVC, the normalized sEMG activity of the working-side MM and balancing-side TA were higher than those of the controls.
Manfredini et al. 2011 (28)	RDC/TMD	Thirty-six subjects with a diagnosis of myofascial pain either without (Ia) or with limited (Ib) opening (24 women, 12 men, mean age 34 ± 9) and an age- and sex-matched group of 36 TMD-free asymptomatic subjects	sEMG assessments of the MM and TA muscles were made at rest and during clenching tasks.	EMG data at rest were not significantly different between myofascial pain patients and asymptomatic subjects, while the EMG potentials were significantly higher during clenching tasks. Symmetry of muscle activity at rest and during clenching tasks did not differ between groups. Fair to excellent accuracy (>0.7) to discriminate between the 2 groups was found only for EMG parameters during clenching tasks. Clenching tasks also showed acceptable sensitivity (77.8–91.7%) and specificity (76.7–86.7%).
Mapelli et al. 2016 (36)	RDC/TMD	Thirty chronic TMD patients (myalgia or/and arthralgia) with DDR (mean age 59.2 ± 52.7) divided into two 15-patient subgroups, with moderate (TMDmo) and severe (TMDse) signs and symptoms and a control group of 15 healthy subjects matched by age (14 women and 1 man)	sEMG measurements of the MM and TA muscles of both sides was recorded during MVC and right and left 15 s unilateral gum chewing tests.	During MVC TMDse group had a significantly lower maximal activity of the TA and MM muscles and larger asymmetry on TA muscles than the control group. During chewing, TMDse patients recruited the balancing side muscles proportionally more than controls, specifically the masseter muscle. When comparing right and left side chewing, the muscles’ recruitment pattern resulted less symmetric in TMD patients, especially in TMDse.
Pires et al. 2018 (37)	RDC/TMD	Seventy-four women with myogenous TMD (myofascial pain (Ia) or myofascial pain with limited mouth opening (Ib), mean age 26.54 ± 2.45) and 30 asymptomatic women as the control (mean age: 25.85 ± 2.57)	sEMG recordings of the right and left TA, MM and suprahyoid muscles were performed during MVC on parafilm.	sEMG potentials were significantly higher in the MM muscles of the control group than the group with myogenous TMD. sEMG values were significantly higher in the MM muscles than the TA muscles in the control group. MPF values of the suprahyoid muscles were significantly higher in the myogenous TMD group than the control group.
Rodrigues et al. 2004 (26)	Clinical exam	Thirty five female volunteers: 19 TMD-pain patients (mean age 23.04 ± 3.5) and 16 clinically normal subjects (mean age 23.3 ± 3.0)	sEMG recordings of the TA and MM muscles were performed at rest and during MVC.	The TMD-P group showed higher EMG activity of the TA and MM muscles at rest compared with the controls. No difference was observed in MMA activity between the groups during MVC.
Santana-Mora et al. 2009 (9)	RDC/TMD	Fifty women with chronic unilateral TMD (both artralgia and myalgia, age 20.46 ± 1.34) and 25 pain-free control subjects (age 20.40 ± 1.32). Twenty five subjects presented with right side TMD and 25 presented with left side TMD.	The EMG recordings of the TA and MM muscles were performed during MVC.	The EMG potentials of the TA and MM muscles for TMD-P patients were significantly lower than those of the control group. Right TA and right MM showed lower EMG activity in the right- than in the left-TMD.
Szyszka-Sommerfeld el al. 2020 (6)	RDC/TMD	Ninety patients divided into 3 groups: a TMD-pain group (30 subjects with myogenous and/or arthrogenous TMD, 16 girls and 14 boys, age 8.8 ± 1.5), a pain-free group (30 subjects with TMD-PF, 14 boys and 16 girls, age 9.0 ± 1.3), and a control group (30 subjects without TMD, 15 girls and 15 boys, age 8.9 ± 1.6)	The EMG potentials of the TA and MM muscles were measured at rest and during MVC in the intercuspal position and on cotton rolls.	Significantly higher rest TA activity was noted in TMD-P subjects compared with that children from the TMD-PF and control groups, as well as in TMD-PF children in relation to those without TMD. The EMG potentials of the TA muscle during MVC were significantly lower in patients with TMD-P than in TMD-PF and control subjects. MM muscle activity at rest in the TMD-P group was significantly greater, and MM muscle EMG potentials during clenching were significantly lower than in controls.
Szyszka-Sommerfeld et al. 2022 (18)	RDC/TMD	Sixty children of both sexes divided into 2 groups: myofascial pain group (30 children with myofascial pain (Group Ia and Ib) and awake bruxism, mean age 9.65 ± 1.25) and control group (30 children without TMD, mean age 9.33 ± 1.42)	The electrical activity of the TA and MM muscles was recorded at rest and during MVC in an intercuspal position and with two 10-mm cotton rolls.	The EMG potentials of the right (R) and left (L) TA and MM muscles at rest were significantly higher in the TMD-P group compared to the control group. During MVC, temporal (RTA) and masseter (LMM, MMmean) muscle EMG activity was significantly lower in children with TMD-P than in subjects with no TMD. Moderate degree of sEMG accuracy (AUC > 0.7) in discriminating between TMD-pain and non-TMD children was observed for TAmean, left MM, and MMmean EMG muscle activity at rest.
Targalia et al. 2008 (38)	RDC/TMD	One hundred and three TMD patients (90 women, mean age 42 ± 16; 13 men, mean age 41 ± 16) subdivided into 3 non-overlapping groups: (a) 25 myogenous; (b) 61 arthrogenous; and (c) 17 psycogenous patients. The control group included 32 subjects matched for sex and age (25 women, 7 men).	sEMG measurements of the right and left MM and TA muscles were performed during MVC in the intercuspal position and on cotton rolls.	The control subjects had the largest standardized EMG muscle activity during MVC, followed by the myogenous and arthrogenous patients; the lowest value was found in the psycogenous patients. Symmetry in the TA muscles was larger in control subjects compared to the TMD patients.
Targalia et al. 2011 (39)	RDC/TMD	Thirty arthrogenous TMD patients (15 men, 15 women, mean age 23.2 ± 3.5) with long lasting pain (more than 6 months), and 20 sex- and age-matched control subjects without signs or symptoms of TMD (mean age 22.6 ± 2.8)	sEMG recordings of the right and left MM and TA muscles were performed during MVC in intercuspal position and on cotton rolls.	During clenching tasks, the TMD patients had larger asymmetry in their TA muscles, larger TA activity relative to MM, and reduced MPFs than the control subjects. No significant difference was observed in standardized total muscle activity between the groups.
Valentino et al. 2021 (40)	DC/TMD	Twenty seven women with chronic TMD myalgia (mTMD, mean age 38.3 ± 12.8) and 18 TMD-free women (mean age 36.2 ± 12.9)	The EMG activity of the TA and MM muscles of both sides were recorded during clenching tasks (MVC in the intercuspal position and on cotton rolls) and while chewing gum on the right and left side.	Women with mTMD had greater muscle work than controls. The EMG activity of TA and MM were similar between right and left sides in both groups. mTMD patients had a significantly greater activity of MM muscles than TMD-free women. No differences between groups were found in chewing rate.

RDC/TMD, Research Diagnostic Criteria for Temporomandibular Disorders; DC/TMD, Diagnostic Criteria for Temporomandibular Disorders; TMD, temporomandibular disorders; TMJ, temporomandibular joint; TMD-P, pain-related TMD; TMD-PF, pain-free TMD; sEMG, surface electromyography; TA, temporal anterior muscle; MM, masseter muscle; MVC, maximal voluntary clenching; AUC, area under ROC curve; MPF, mean power frequency; DDR, disc displacement with reduction.

The quality assessment results for each study are summarized in Table 2. The Cohen’s Kappa coefficient for the agreement between the authors was calculated as 0.92. Global quality rating using the EPHPP Quality Assessment Tool for Quantitative Studies for most of the studies (*n* = 10) was weak.

**Table 2:** The quality assessment of the studies included (EPHPP instrument).

Authors, year	Selection bias	Study design	Confounders	Blinding	Data collection methods	Withdrawals and dropouts	Global ratings
Berni et al., 2015 (17)	Moderate	Moderate	Moderate	Moderate	Strong	Weak	Moderate
Bodéré et al., 2005 (33)	Moderate	Moderate	Moderate	Weak	Moderate	Weak	Weak
Glaros et al., 1997 (34)	Moderate	Moderate	Moderate	Weak	Moderate	Weak	Weak
Li et al., 2016 (35)	Weak	Moderate	Moderate	Weak	Moderate	Weak	Weak
Manfredini et al., 2011 (28)	Moderate	Moderate	Moderate	Moderate	Moderate	Weak	Moderate
Mapelli et al., 2016 (36)	Moderate	Moderate	Moderate	Moderate	Moderate	Weak	Moderate
Pires et al., 2018 (37)	Moderate	Moderate	Moderate	Weak	Moderate	Weak	Weak
Rodrigues et al., 2004 (26)	Weak	Moderate	Moderate	Weak	Moderate	Weak	Weak
Santana-Mora et al., 2009 (9)	Strong	Moderate	Moderate	Weak	Moderate	Weak	Weak
Szyszka-Sommerfeld et al., 2020 (6)	Moderate	Moderate	Moderate	Weak	Moderate	Weak	Weak
Szyszka-Sommerfeld et al., 2022 (18)	Moderate	Moderate	Moderate	Weak	Strong	Weak	Weak
Targalia et al., 2008 (38)	Moderate	Moderate	Moderate	Moderate	Moderate	Weak	Moderate
Targalia et al., 2011 (39)	Weak	Moderate	Moderate	Weak	Moderate	Weak	Weak
Valentino et al., 2021 (40)	Moderate	Moderate	Moderate	Weak	Moderate	Weak	Weak

Criteria for global ratings: strong = no weak ratings; moderate = one weak rating; weak = two or more weak ratings.

Two of the included articles focused on evaluating MMA in children and adolescents with TMD-P (6, 18). Adults were included in remaining 12 studies.

The Research Diagnostic Criteria for Temporomandibular Disorders were the most frequently used criteria to diagnose and categorize TMD (*n* = 9), followed by clinical examination (*n* = 4), and the DC/TMD (*n* = 1). Within these criteria, muscle disorders (myofascial pain, myalgia) were the most frequently studied subgroup (*n* = 7) (17, 18, 26, 28, 34, 37, 40), followed by mixed TMD (*n* = 6) - myalgia and arthralgia diagnoses (6, 9, 35, 36); myogenous, arthrogenous and psycogenous pain groups (38); myalgia and neuropatic pain groups (33). Arthrogenous TMD were the least reported (*n* = 1) (39).

Masticatory muscle activity was reported using sEMG under different conditions: (a) resting position (b) clenching, including MVC (maximal voluntary clenching) in the intercuspal position and MVC on cotton rolls or parafilm (c) and chewing. The masseter (MM) and temporal anterior (TA) muscles were assessed in all studies analyzed, while the suprahyoid muscles were assessed in 2 studies.

Out of the 14 studies that were reviewed, 13 studies conducted the processing and analysis of the EMG signal based on the time-domain. One study, however, performed both time-domain and frequency-domain analyses (37).

Masseter and temporal muscle activity at rest was assessed in 7 studies. In 6 of them reported significantly greater MM and TA activity in the TMD-P group compared to a healthy non-TMD group (6, 17, 18, 26, 33, 34). In contrast, one study found no significant differences in the MM and TA electromyographical activity between myofascial pain subjects and asymptomatic controls (28).

Masseter muscle EMG activity during MVC was measured in 12 studies. Of these, 8 reported significantly lower MM activity in the TMD-P group compared to asymptomatic non-TMD patients (6, 9, 17, 18, 35–38) and 2 reported higher MM electrical activity in the pain-related TMD group (28, 40). Two studies found no significant differences in the MM electrical potentials between pain-related TMD patients and asymptomatic control subjects (26, 39). Twelve studies examined TA muscle electrical activity during MVC. Most of them (*n* = 6) showed lower EMG potentials in the pain-related TMD group compared to the control group (6, 9, 18, 35, 36, 38) and one observed higher EMG activity of the TA muscles in the TMD-pain group (28). Five studies found no significant differences in the temporal muscle EMG activity during MVC between TMD-pain patients and asymptomatic controls (17, 26, 37, 39, 40).

Two studies examined masseter and temporal muscle electrical potentials during chewing (36, 40). One study found no differences in the MM and TA activity between the TMD-P group and the control group (40). A single study observed that individuals with TMDse exhibited greater recruitment of muscles on the balancing side during chewing, particularly in the masseter muscle, when compared to control subjects (36).

The EMG activity of the suprahyoid muscles was measured in 2 studies (17, 38). The results showed significantly greater EMG activity in TMD-P patients during MVC on parafilm in comparison to the control group.

Four studies evaluated the diagnostic efficiency of surface electromyography in assessing patients with TMD-P (17, 18, 28, 34). In one study, sEMG was found to have moderate accuracy in differentiating between children with pain-related TMD and those without TMD, specifically for MMmean, left MM and TAmean normalized EMG activity at rest (18). Another two studies investigated the diagnostic utility of surface electromyography based on raw EMG values for the diagnosis of adults with myofascial pain (17, 28). One study reported moderate accuracy of sEMG in diagnosing TMD-P and healthy non-TMD individuals at rest. The authors stated that the surface electromyography is a valuable additional tool for diagnosis of TMD patients with myofascial pain of the masticatory muscles (17). In contrast, one study found that fair to excellent accuracy in discrimination between patients diagnosed with myofascial pain and non-TMD controls was obtained only for EMG values during teeth clenching tasks. However, the authors warned against the potential risk of overdiagnosis and overtreatment when using sEMG as a diagnostic tool for myogenous TMD (28). In addition, another study showed that the EMG data at rest provided little support for precisely distinguishing between TMD-pain patients and asymptomatic controls (34).

## 4. Discussion

This systematic review presents relevant findings on masticatory muscle activity during various tasks in individuals with pain-related TMD, as well as the diagnostic utility of sEMG in assessing patients with TMD-P. Fourteen studies examining the MMA in the rest position, during MVC and chewing were included in the review.

It was shown that electromyographical activity of the masticatory muscles differed between the TMD-pain and non-TMD groups. In addition, the direction of changes in MMA varied depending on the task studied. During the evaluation of pain-related TMD subjects using sEMG, the tasks that were most frequently analyzed included resting muscle activity and muscle activity during MVC. Most studies showed that the EMG activity of the MM and TA muscles at rest in TMD-P subjects was higher than in the asymptomatic controls (6, 17, 18, 26, 33, 34), while the MM and TA muscles were less active in pain-related subjects than in the control group during MVC (6, 9, 18, 35, 36, 38). This observation suggests that the presence of pain may result in reduction in muscle activity and restriction of movement patterns as a protective mechanism to prevent injury (41, 42).

The symmetry of muscle activity between sides is another crucial factor to consider. A number of studies included in this systematic review found a higher level of asymmetry in pain-related TMD groups compared with healthy, pain-free control groups (36, 38, 39), while others found no difference in the symmetry of muscle activity between TMD-P patients and asymptomatic controls (28).

Clinically, it is crucial to note that alterations in the pattern of muscle activity in individuals with TMD-P can impact muscle fatigue and, therefore, muscle function (43). Muscle activity testing should include examination of specific muscle groups in certain tasks to properly recognize abnormalities. Normalizing muscle activity and improving muscle function are essential aspects of developing effective treatment protocols for TMD-P patients.

sEMG is a widely used non-invasive technique that has found application in the diagnosis of patients with general muscle disorders, neuromuscular diseases or diseases affecting neuromuscular performance (18). In dentistry, sEMG plays an important role in the assessment of painful and non-painful conditions of TMDs, dystonia, head and neck muscle diseases, cranial nerve lesions, as well as seizures and sleep disorders (44). Adequate quality of the EMG assessment and reporting is essential to ensure reliable evaluation of patients with TMD. It should be noted that one of the main disadvantages of surface electromyography is its sensitive to impedance imbalances, which may reduce the accuracy of the EMG recordings (19, 27). Problems with the reproducibility of sEMG related to technical artifacts (instrumental noise), anatomical variations such as facial type, age, gender, thickness of subcutaneous fat, as well as muscle cross-talk, may hinder its clinical validity (4). The reproducibility of sEMG is also debatable due to the different inter-electrode distances and their various positioning over muscles. Therefore, special attention should be paid to establishing a fixed inter-electrode distances and creating a standardized protocol for surface electrode placement. In this context, it should be emphasized that the precision of EMG outcomes can be greatly influenced by a number of factors, including technical aspects, such as positioning of electrodes, signal processing, as well as the particular hardware and software used (19, 27). In the included studies, EMG recordings of the subjects were performed using a variety of EMG devices with different technical parameters, including: the DAB-Bluetooth Instrument (Zebris Medical GmbH, Germany) (6, 18), the BIO-EMG 1000 electromyograph (Lynx Tecnologia Eletrônica Ltda, São Paulo, SP, Brazil) (17, 26, 37), the K6 Diagnostic System (Myotronics Inc., Seattle, WA, USA) (28), the computerized instrument (Freely, De Götzen srl; Legnano, Milano, Italy) (38, 39), the Nicolet Viking Select electrodiagnostic system (Nicolet Biomedical, Madison, WI, USA) (9, 33), the EA-1, J&J Instruments (Poulsboro, WA, USA) (34), the EMG system (TMJOINT, BTS, SpA, Garbagnate Milanese, Italy) (36, 40), the BioEMG III with a BioPak Measurement System, version 6.0 (Bioresearch Associates, Inc., Milwaukee, WI, USA) (35). Prior to the electrodes placement, the patient’s skin surface was cleaned to reduce impedance (6, 9, 17, 18, 34, 36, 37, 40). Different types of surface electrodes were placed over the muscle belly of the masseter, anterior temporal and suprahyoid muscles in specific positions, and then all experimental tasks were performed. During the EMG recordings, the subjects sat in a chair with natural head position. To evaluate the EMG data, the researchers used various parameters to analyze the electromyographic signal in TMD-P subjects. Data processing was performed in the amplitude domain with normalized (6, 18, 35, 36, 38–40) or non-normalized data (9, 17, 26, 28, 33, 34) and/or in the frequency domain (37). In this context, it should be noted that the normalization process is essential to ensure intercomparisons and further data analysis. Normalized EMG data will offer insight into the impact of occlusion on neuromuscular activity, while disregarding individual variations such as anatomical variances, physiological and psychological state, and others. To standardize the interpretation of muscle electrical potentials, normalization techniques are used to compare them with reference values obtained during standardization recordings. In the standardization recording, among the various protocols, MVC on two cotton rolls positioned on mandibular molars is now commonly used (6, 18, 21, 36, 38–40, 45).

It should also be emphasized that several restraints of sEMG, including its time-consuming nature, the need of specialized equipment, and the need of proper training and calibration of the examiner may limit the use of sEMG in clinical settings. However, the advantages of sEMG such as its ease of use, availability, and non-invasive and painless nature, partially compensate for the aforementioned limitations (19). For these reasons, the method can also be used in children and adolescents (6, 18).

In light of the above, it is also important to evaluate the diagnostic utility of surface electromyography in the diagnosis of pain-related TMD. The utilization of sEMG as a tool for evaluating patients with TMD-pain diagnosis versus RDC/TMD as the gold standard is debatable due to the significant variability in results (17, 18, 27, 28). While some authors have suggested that sEMG can be used as an additional diagnostic tool for identifying TMD pain (17, 18), others have argued that electromyography may not be useful for this purpose (28, 34). These findings encourage the search for other measurable instrumental diagnostic methods that allow objective and quantitative analysis of masticatory muscle function and may be useful in the assessment of TMD-P, such as thermography, kinesiography or pressure algometry (28, 46–52). The advantages of thermography, including its non-invasiveness, lack of ionizing radiation, and relatively low cost, are sufficient to recommend its use among the supplementary tools employed in TMD diagnosis. However, the validity of the use of thermography in the evaluation of TMD is still under investigation (46, 47, 50). Some studies have confirmed the diagnostic utility of thermography in identifying patients with TMD (46, 51), while other researches have observed low accuracy of infrared thermography analysis in differentiating between subjects with myogenous TMD and asymptomatic controls (50). An important limitation of using this method in clinical practice is that there is no standardized protocol for measuring masticatory muscle temperature using infrared thermography. Standardization of all protocols requires ensuring that all possible thermal changes related to the image acquisition room and patient habits do not interfere with the acquisition of data. Therefore, the need of control the measurement conditions, as well as problems with complete objectification of the results may limit the use of thermography in the assessment of TMD (47). The diagnostic utility of kinesiography has also been questioned. Low accuracy using kinesiography recordings in identifying subjects with myofascial pain in the masticatory muscles has been reported (28). The lack of normative values on which to base discriminatory power between TMD patients and asymptomatic subjects is a major limitation to conclusively assessing the validity and application of this method in a clinical practice. Similarly, pressure algometry is a non-invasive and easy to use method to assess TMD, but because of the specific nature of the examination, this technique is also dependent on a number of factors which may limited its application. A key element is maintaining consistent test conditions. Among the main factors that are particularly important in this regard are the invariability of the position of the algometer in relation to the structures under examination, the dynamics of the pressure exerted, the area to which pressure is applied, and the differences between algometers (48). While some authors have confirmed the high diagnostic value of pressure algometry in differentiating TMD cases from controls (48, 52), others report low accuracy of pressure algometry in diagnosis of myofascial pain of the masticatory muscles (49).

This systematic review presents some limitations that should be acknowledged: (a) most of the included studies were of weak quality according to the EPHPP tool; (b) only four articles included in this review focused on the diagnostic utility in differentiating between TMD-P and asymptomatic control subjects; (c) the use of different EMG devices and various parameters to analyze the EMG signal may affect the results among the included studies; (d) we should also be aware that the differences in study groups characteristics such as gender, age, TMD subgroup may affect the EMG results; (e) as the reliability and validity of sEMG largely depends on biological, instrumental and technical factors, future studies need to investigate the quality of EMG testing and reporting procedures using a standardized framework.

## 5. Conclusion

This systematic review comprehensively examines changes in masticatory muscle activity in pain-related TMD subjects using surface electromyography. Differences were found in masticatory muscle activity in the TMD-pain population compared to a healthy control group during various tasks. The diagnostic efficacy of surface electromyography in assessing individuals with pain-related TMD remains unclear.

## Author contributions

LS-S conception and design of the study, manuscript writing, and realization of figures and tables. LS-S and MS-D literature review and article selection. GS, MS-D, and KW participation in the writing of the manuscript. All authors contributed to the article and approved the submitted version.

## Conflict of interest

The authors declare that the research was conducted in the absence of any commercial or financial relationships that could be construed as a potential conflict of interest.

## Publisher’s note

All claims expressed in this article are solely those of the authors and do not necessarily represent those of their affiliated organizations, or those of the publisher, the editors and the reviewers. Any product that may be evaluated in this article, or claim that may be made by its manufacturer, is not guaranteed or endorsed by the publisher.

## References

[1] Orzeszek S Waliszewska-Prosol M Ettlin D Seweryn P Straburzynski M Martelletti P. Efficiency of occlusal splint therapy on orofacial muscle pain reduction: a systematic review. BMC Oral Health. (2023) 23:180. doi: 10.1186/s12903-023-02897-0, PMID: 36978070 PMC10053140

[2] Li DTS Leung YY. Temporomandibular disorders: current concepts and controversies in diagnosis and management. Diagnostics (Basel). (2021) 11:459. doi: 10.3390/diagnostics11030459, PMID: 33800948 PMC8000442

[3] Scrivani SJ Keith DA Kaban LB. Temporomandibular disorders. N Engl J Med. (2008) 359:2693–705. doi: 10.1056/NEJMra0802472 19092154

[4] Dworkin SF LeResche L. Research diagnostic criteria for temporomandibular disorders: review, criteria, examinations and specifications, critique. J Craniomand Disord. (1992) 6:301–55. 1298767

[5] De Leeuw R Klasser GD. Orofacial Pain - Guidelines for Assessment, Diagnosis and Management. Chicago: Quintessence (2013).

[6] Szyszka-Sommerfeld L Machoy M Lipski M Woźniak K. Electromyography as a means of assessing masticatory muscle activity in patients with pain-related temporomandibular disorders. Pain Res Manag. (2020) 2020:9750915. doi: 10.1155/2020/9750915, PMID: 32855751 PMC7443041

[7] Khawaja SN McCall W Jr Dunford R Nickel JC Iwasaki LR Crow HC. Infield masticatory muscle activity in subjects with pain-related temporomandibular disorders diagnoses. Orthod Craniofac Res. (2015) 18:137–45. doi: 10.1111/ocr.12077, PMID: 25865542 PMC4396706

[8] Alkhubaizi Q Khalaf ME Faridoun A. Prevalence of temporomandibular disorder-related pain among adults seeking dental care: a cross-sectional study. Int J Dent. (2022) 2022:3186069. doi: 10.1155/2022/3186069, PMID: 36105380 PMC9467697

[9] Santana-Mora U Cudeiro J Mora-Bermúdez MJ Rilo-Pousa B Ferreira-Pinho JC Otero-Cepeda JL. Changes in EMG activity during clenching in chronic pain patients with unilateral temporomandibular disorders. J Electromyogr Kinesiol. (2009) 19:e543–9. doi: 10.1016/j.jelekin.2008.10.002, PMID: 19041265

[10] LeResche L. Epidemiology of temporomandibular disorders: implications for the investigation of etiologic factors. Crit Rev Oral Biol Med. (1997) 8:291–305. doi: 10.1177/10454411970080030401, PMID: 9260045

[11] Valesan LF Da-Cas CD Réus JC Denardin ACS Garanhani RR Bonotto D. Prevalence of temporomandibular joint disorders: a systematic review and meta-analysis. Clin Oral Investig. (2021) 25:441–53. doi: 10.1007/s00784-020-03710-w, PMID: 33409693

[12] Schiffman E Ohrbach R Truelove E Look J Anderson G Goulet JP. Diagnostic criteria for temporomandibular disorders (DC/TMD) for clinical and research applications: recommendations of the international RDC/TMD consortium network* and orofacial pain special interest Groupdagger. J Oral Facial Pain Headache. (2014) 28:6–27. doi: 10.11607/jop.1151, PMID: 24482784 PMC4478082

[13] Wieckiewicz M Grychowska N Wojciechowski K Pelc A Augustyniak M Sleboda A. Prevalence and correlation between TMD based on RDC/TMD diagnoses, oral parafunctions and psychoemotional stress in polish university students. Biomed Res Int. (2014) 2014:472346. doi: 10.1155/2014/472346, PMID: 25121100 PMC4119893

[14] Wieckiewicz M Paradowska-Stolarz A Wieckiewicz W. Psychosocial aspects of bruxism: the paramount factor influencing teeth grinding. Biomed Res Int. (2014) 2014:469187. doi: 10.1155/2014/469187, PMID: 25101282 PMC4119714

[15] Medllicott MS Harris SR. A systematic review of the effectiveness of exercise, manual therapy, electrotherapy, relaxation training, and biofeedback in the management of temporomandibular disorder. Phys Ther. (2006) 86:955–73. doi: 10.1093/ptj/86.7.955, PMID: 16813476

[16] Gonzalez YM Greener CS Mohl ND. Technological devices in the diagnosis of temporomandibular disorders. Oral Maxillofac Surg Clin North Am. (2008) 20:211–20. doi: 10.1016/j.coms.2007.12.006, PMID: 18343326

[17] Berni KC Dibai-Filho AV Pires PF Rodrigues-Bigaton D. Accuracy of the surface electromyography RMS processing for the diagnosis of myogenous temporomandibular disorder. J Electromyogr Kinesiol. (2015) 25:596–602. doi: 10.1016/j.jelekin.2015.05.004, PMID: 26054969

[18] Szyszka-Sommerfeld L Sycińska-Dziarnowska M Budzyńska A Woźniak K. Accuracy of surface electromyography in the diagnosis of pain-related temporomandibular disorders in children with awake bruxism. J Clin Med. (2022) 11:1323. doi: 10.3390/jcm11051323, PMID: 35268414 PMC8911396

[19] Woźniak K Piątkowska D Lipski M Mehr K. Surface electromyography in orthodontics - a literature review. Med Sci Monit. (2013) 19:416–23. doi: 10.12659/MSM.883927, PMID: 23722255 PMC3673808

[20] Al-Saleh MA Armijo-Olivo S Flores-Mir C Thie NM. Electromyography in diagnosing temporomandibular disorders. J Am Dent Assoc. (2012) 143:351–62. doi: 10.14219/jada.archive.2012.0177 22467695

[21] De Felício CM Ferreira CL Medeiros AP Rodriques Da Silva MA Targalia GM Sforza C. Electromyographic indices, orofacial myofunctional status and temporomandibular disorders severity: a correlation study. J Electromyogr Kinesiol. (2012) 22:266–72. doi: 10.1016/j.jelekin.2011.11.013, PMID: 22206640

[22] Ferrario VF Serrao G Dellavia C Caruso E Sforza C. Relationship between the number of occlusal contacts and masticatory muscle activity in healthy young adults. Cranio. (2002) 20:91–8. doi: 10.1080/08869634.2002.11746196, PMID: 12002835

[23] Ferrario VF Tartaglia GM Galletta A Grassi GP Sforza C. The influence of occlusion on jaw and neck muscle activity: a surface EMG study in healthy young adults. J Oral Rehabil. (2006) 33:341–8. doi: 10.1111/j.1365-2842.2005.01558.x, PMID: 16629892

[24] Dinsdale A Liang Z Thomas L Treleaven J. Is jaw muscle activity impaired in adults with persistent temporomandibular disorders? A systematic review and meta analysis. J Oral Rehabil. (2021) 48:487–516. doi: 10.1111/joor.13139, PMID: 33369753

[25] Nielsen L McNeill C Danzig W Goldman S Levy J Miller AJ. Adaptation of craniofacial muscles in subjects with craniomandibular disorders. Am J Orthod Dentofac Orthop. (1990) 97:20–34. doi: 10.1016/S0889-5406(05)81705-6, PMID: 2296935

[26] Rodrigues D Siriani AO Berzin F. Effect of conventional TENS on pain and electromyographic activity of masticatory muscles in TMD patients. Br Oral Res. (2004) 18:290–5. doi: 10.1590/s1806-83242004000400003, PMID: 16089258

[27] Szyszka-Sommerfeld L Machoy M Lipski M Woźniak K. The diagnostic value of electromyography in identifying patients with pain-related temporomandibular disorders. Front Neurol. (2019) 10:180. doi: 10.3389/fneur.2019.00180, PMID: 30891001 PMC6411686

[28] Manfredini D Cocilovo F Favero L Ferronato G Tonello S Guarda-Nardini L. Surface electromyography of jaw muscles and kinesiographic recordings: diagnostic accuracy for myofascial pain. J Oral Rehabil. (2011) 38:791–9. doi: 10.1111/j.1365-2842.2011.02218.x, PMID: 21480942

[29] Santana-Mora U López-Ratón M Mora MJ Cadarso-Suárez C López-Cedrún J Santana-Penín U. Surface raw electromyography has a moderate discriminatory capacity for differentiating between healthy individuals and those with TMD: a diagnostic study. J Electromyogr Kinesiol. (2014) 24:332–40. doi: 10.1016/j.jelekin.2014.03.001, PMID: 24698167

[30] Page MJ McKenzie JE Bossuyt PM Boutron I Hoffmann TC Mulrow CD. The PRISMA 2020 statement: an updated guideline for reporting systematic reviews. BMJ. (2021) 372:n71. doi: 10.1136/bmj.n71, PMID: 33782057 PMC8005924

[31] Sackett DL Strauss SE Richardson WS Rosenberg W Haynes BR. Evidence Based Medicine: How to Practice and Teach EBM. 2nd ed. Philadelphia: Elsevier Churchill Livingstone (2000).

[32] Thomas BH Ciliska D Dobbins M Micucci S. A process for systematically reviewing the literature: providing the research evidence for public health nursing interventions. Worldviews Evid-Based Nurs. (2004) 1:176–84. doi: 10.1111/j.1524-475X.2004.04006.x 17163895

[33] Bodéré C Hack Téa S Giroux-Metges MA Woda A. Activity of masticatory muscles in subjects with different orofacial pain conditions. Pain. (2005) 116:33–41. doi: 10.1016/j.pain.2005.03.011, PMID: 15927390

[34] Glaros AG Glass EG Brockman D. Electromyographic data from TMD patients with myofascial pain and from matched control subjects: evidence for statistical, not clinical, significance. J Orofac Pain. (1997) 11:125–9. PMID: 10332318

[35] Li BY Zhou LJ Guo SX Zhang Y Lu L Wang MQ. An investigation on the simultaneously recorded occlusion contact and surface electromyographic activity for patients with unilateral temporomandibular disorders pain. J Electromyogr Kinesiol. (2016) 28:199–207. doi: 10.1016/j.jelekin.2015.11.002, PMID: 26643794

[36] Mapelli A Zanandréa Machado BC Giglio LD Sforza C De Felício CM. Reorganization of muscle activity in patients with chronic temporomandibular disorders. Arch Oral Biol. (2016) 72:164–71. doi: 10.1016/j.archoralbio.2016.08.022, PMID: 27597536

[37] Pires PF Rodrigues-Bigaton D. Evaluation of integral electromyographic values and median power frequency values in women with myogenous temporomandibular disorder and asymptomatic controls. J Bodyw Mov Ther. (2018) 22:720–6. doi: 10.1016/j.jbmt.2017.09.001, PMID: 30100303

[38] Tartaglia GM Rodrigues M da Silva MA Bottini S Sforza C Ferrario VF. Masticatory muscle activity during maximum voluntary clench in different research diagnostic criteria for temporomandibular disorders (RDC/TMD) groups. Man Ther. (2008) 13:434–40. doi: 10.1016/j.math.2007.05.011, PMID: 17643338

[39] Tartaglia GM Lodetti G Paiva G De Felicio CM Sforza C. Surface electromyographic assessment of patients with long lasting temporomandibular joint disorder pain. J Electromyogr Kinesiol. (2011) 21:659–64. doi: 10.1016/j.jelekin.2011.03.003, PMID: 21463956

[40] Valentino R Cioffi I Vollaro S Cimino R Baiano R Michelotti A. Jaw muscle activity patterns in women with chronic TMD myalgia during standardized clenching and chewing tasks. Cranio. (2021) 39:157–63. doi: 10.1080/08869634.2019.1589703, PMID: 30896353

[41] Nickel JC Iwasaki LR Walker RD McLachlan KR McCall WD Jr. Human masticatory muscle forces during static biting. J Dent Res. (2003) 82:212–7. doi: 10.1177/154405910308200312, PMID: 12598551

[42] Sessle BJ. Acute and chronic craniofacial pain: brainstem mechanisms of nociceptive transmission and neuroplasticity, and their clinical correlates. Crit Rev Oral Biol Med. (2000) 11:57–91. doi: 10.1177/10454411000110010401, PMID: 10682901

[43] Ries LGK Graciosa MD Soares LP Sperandio FF Santos GM Degan VV. Effect of time of contraction and rest on the masseter and anterior temporal muscles activity in subjects with temporomandibular disorder. Codas. (2016) 28:155–62. doi: 10.1590/2317-1782/201620150112, PMID: 27191879

[44] Nishi SE Basri R Alam MK. Uses of electromyography in dentistry: an overview with Meta-analysis. Eur J Dent. (2016) 10:419–25. doi: 10.4103/1305-7456.184156, PMID: 27403065 PMC4926600

[45] Ferrario VF Sforza C Colombo A Ciusa V. An electromyographic investigation of masticatory muscles symmetry in normo-occlusion subjects. J Oral Rehabil. (2000) 27:33–40. doi: 10.1046/j.1365-2842.2000.00490.x PMID: 10632841

[46] Woźniak K Szyszka-Sommerfeld L Trybek G Piątkowska D. Assessment of the sensitivity, specificity, and accuracy of thermography in identifying patients with TMD. Med Sci Monit. (2015) 21:1485–93. doi: 10.12659/MSM.893863, PMID: 26002613 PMC4451701

[47] Machoy M Szyszka-Sommerfeld L Rahnama M Koprowski R Wilczyński S Woźniak K. Diagnosis of temporomandibular disorders using Thermovision imaging. Pain Res Manag. (2020) 2020:1–8. doi: 10.1155/2020/5481365 PMC7685803 33282037

[48] Więckiewicz W Woźniak K Piątkowska D Szyszka-Sommerfeld L Lipski M. The diagnostic value of pressure algometry for temporomandibular disorders. Biomed Res Int. (2015) 2015:575038. doi: 10.1155/2015/575038, PMID: 25883964 PMC4391694

[49] Farella M Michelotti A Steenks MH Romeo R Cimino R Bosman F. The diagnostic value of pressure algometry in myofascial pain of the jaw muscles. J Oral Rehabil. (2000) 27:9–14. doi: 10.1046/j.1365-2842.2000.00526.x, PMID: 10632838

[50] Dibai Filho AV Packer AC Costa AC Rodrigues-Bigaton D. Accuracy of infrared thermography of the masticatory muscles for the diagnosis of myogenous temporomandibular disorder. J Manip Physiol Ther. (2013) 36:245–52. doi: 10.1016/j.jmpt.2013.04.007, PMID: 23706912

[51] McBeth SB Gratt BM. Thermographic assessment of temporomandibular disorders symptomology during orthodontic treatment. Am J Orthod Dentofac Orthop. (1996) 109:481–8. doi: 10.1016/S0889-5406(96)70132-4, PMID: 8638592

[52] Bernhardt O Schiffman EL Look JO. Reliability and validity of a new fingertip-shaped pressure algometer for assessing pressure pain thresholds in the temporomandibular joint and masticatory muscles. J Orofac Pain. (2007) 21:29–38. PMID: 17312639

